# The effect of counseling, antiretroviral therapy and relationship on disclosing HIV positive status to sexual partner among adult HIV patients in Ethiopia: A systematic review and meta-analysis

**DOI:** 10.1371/journal.pone.0249887

**Published:** 2021-04-22

**Authors:** Melaku Yalew, Bezawit Adane, Bereket Kefale, Yitayish Damtie, Sisay Eshete Tadesse, Asressie Molla

**Affiliations:** 1 Department of Reproductive and Family Health, School of Public Health, College of Medicine and Health Sciences, Wollo University, Dessie, Ethiopia; 2 Department of Epidemiology and Biostatistics, School of Public Health, College of Medicine and Health Sciences, Wollo University, Dessie, Ethiopia; 3 Department of Nutrition and Dietetics, School of Public Health, College of Medicine and Health Sciences, Wollo University, Dessie, Ethiopia; 1. IRCCS Neuromed 2. Doctors with Africa CUAMM, ITALY

## Abstract

**Background:**

Human Immunodeficiency Virus (HIV) is continued as a major public health problem, especially in developing countries. Therefore, this study aimed to estimate the effect of counseling, antiretroviral therapy (ART) and relationship on disclosing HIV positive status to sexual partner among adult HIV patients in Ethiopia.

**Methods:**

The Preferred Reporting Items for Systematic Review and Meta-analysis (PRISMA) was used during this review. The study included both published and unpublished studies which were conducted in Ethiopia until the end of 2019. Different electronic databases (PubMed, Cochrane library, CINAHL, Global Health, HINARI and Google scholar) were searched. Data were extracted in Microsoft Excel sheet and STATA/SE 14 was used to meta-analysis. I^**2**^ and Egger test statistics were used to test heterogeneity and publication bias respectively.

**Results:**

Twenty-two articles with 8,873 adult HIV infected peoples were included in this systematic review and meta-analysis. The pooled magnitude of disclosing HIV status to sexual partner was 74.63% [95% CI: (67.79, 81.47)]. Counseled [AOR = 4.96, 95% CI: (2.87, 8.55)], ART initiated [AOR = 4.78, 95% CI: (3.84, 5.94)] and who had a smooth relationship before HIV testing [AOR = 6.82, 95% CI: (3.49, 13.33)] were significantly associated with disclosing HIV status to sexual partner.

**Conclusions:**

Disclosing HIV positive status to sexual partner in Ethiopia was low as the government invested in partner notification. Counseling, ART initiation and smooth relationship before HIV testing were significantly associated with disclosing HIV status to sexual partner. The government needs to strengthen pre and post HIV test counseling even after treatment started to increase disclosing status.

**Registration:**

The protocol of this systematic review and meta-analysis was registered in the PROSPERO with a specific registration number: **CRD42020161276**; https://clinicaltrials.gov/.

## Introduction

Globally, an estimated 36.8 million people were living with HIV, of which the burden was high in Sub-Saharan Africa [1,2]. Disclosure of HIV-positive status is a willingness to reveal seropositive status to others or it is communicating potentially stigmatizing information that had previously been kept hidden in order to increase one’s psychological well-being and to preserve the quality of relationships [3]. Although disclosure has several benefits, deciding to disclose even to sexual partner was a big challenge for individuals infected with HIV [4].

Several studies have revealed that individuals diagnosed with HIV infection continue to have unprotected sex without informing their sexual partners, who may be of negative or unknown serostatus [5]. The magnitude of disclosing HIV positive status was ranged from 16.7 to 86% in developing countries [6]. Disclosing HIV status is an important component of HIV prevention as it may motivate partners to know their HIV status. Disclosure has been shown to result in better adherence to therapy, less anxiety and good CD4 recovery following antiretroviral therapy (ART). A recent study showed that serostatus disclosure reduced the risk of HIV transmission by 17.9% to 40.6% [7,8]. Not only this but also, non-disclosure significantly associated to develop depression [9].

Factors that determine disclosure or non-disclosure of HIV status were socio-demographic factors [10–12], psycho-social, cognitive and behavioral factors [9,13–18]. The Sustainable Development Goal aims to end the epidemics of AIDS by the end of the year 2030 [19,20]. There are different strategies (Voluntary Counseling and Testing and Provider initiated testing and counseling) tried so far to prevent HIV transmission in Ethiopia. Within these programs, the emphasis is placed on the importance of disclosing HIV status among HIV-infected clients, particularly to their sexual partners [21].

Disclosing HIV status to sexual partner was somewhat investigated in a different parts of Ethiopia [22–33]. Even, a systematic review and meta-analysis was conducted [34]. But, it was restricted to two factors (knowing a partner’s HIV status and History of discussion on HIV related issues) was considered as a predictor. In addition, the study only considered twelve studies in systematic review and meta-analysis. As each study brought inconsistent and inconclusive findings and there is no single national representative figure about the effect of counseling, ART and relationship on disclosing HIV positive status [35,36], this study will generate very crucial evidence that may require urgent action for program planners or policymakers. So, this study aimed to estimate the effect of counseling, antiretroviral therapy and relationship on disclosing HIV positive status to sexual partner among adult HIV patients in Ethiopia.

## Methods

### Search strategy and registration

The study was designed based on the Preferred Reporting Items for Systematic Reviews and Meta-Analysis Protocols (PRISMA-P 2009) (S1 File) [37]. The protocol of this systematic review and meta-analysis was registered in the PROSPERO, international prospective register of systematic reviews with specific registration number: **CRD42020161276.** The study included both published and unpublished articles which were conducted until the end of December 31, 2019 from different databases: (PubMed, Cochrane library, CINAHL, Global Health, HINARI and Google scholar). All potential articles were searched by using a combination of keywords/indices like; “prevalence”, “magnitude”, “proportion”, “determinants”, "determinant factors", "risk factors", “risks”, "factors associated", "associated factors", “predictors”, “disclosure”, “disclosing”, “expose”, "truth disclosure", "self-disclosing", "self-disclosure", "HIV infected", "HIV patients", "AIDS patients", “spouse”, "sexual partner", “friends” which were developed according to Medical Subject Headings (MeSH). These all key terms were searched by a combination of Boolean operators “AND” or “OR” as appropriate and the search was done by two authors independently (MY and BK).

### Inclusion and exclusion criteria

#### Inclusion criteria

- Population: Studies conducted among adult HIV positive patients in Ethiopia.
- Setting: Conducted at either facility or community based.
- Outcome: Studies conducted disclosing HIV positive status to sexual partner.
- Publication: Either published in peer-reviewed journals or unpublished studies.
- Time frame: All studies irrespective of data collection and publication year until the end of 2019.
- Language: Studies published only in English language were included in this review.

#### Exclusion criteria

- Studies in which the outcome did not clearly reported
- Studies repeatedly published
- Studies which were pure qualitative were excluded from systematic review and meta-analysis.

### Outcome measurement

The outcome variable (disclosing HIV positive status to sexual partner) was measured using “YES” or “NO” questions. It was measured as “YES” if a participant disclosed his/her HIV positive status to his/her most recent sexual partner and “NO” if didn’t.

### Study quality appraisal and data extraction

Articles identified in all databases were exported to Endnote X8 and duplicate files were excluded. The remaining articles were independently screened by two groups (SET and AM) for inclusion in the full-text appraisal. The differences between reviewers were managed through discussion and further disagreement was handled by the third group reviewer (YD). The quality of articles was assessed using Joana Brigg’s Institute (JBI) critical appraisal checklist [38,39]. Two independent authors (BA and MY) assessed the quality of the articles and the differences in the scales result was settled by taking the average result of both reviewers.

Data were extracted using Microsoft excel 2010 sheet and it was conducted in two steps for primary and secondary objective. The data extraction sheet contained the following list of variables for the first objective: authors name followed by initials, year of study, year of publication, study setting, study design, sample size, response rate, quality score, sex of participants, region, study finding (magnitude of disclosing HIV status). For the second objective, first, those studies who reported at least one of those factors as predictors were identified. For each predictor (to calculate the odds ratio), the data were extracted from the primary studies in the form of two by two tables sequentially labeled as A, B, C, D representing the four cells. Two authors (MY and BA) extract the data for both objectives and any disagreements between the two reviewers were solved through discussion.

### Data synthesis and statistical analysis

The descriptive characteristics of the included studies were presented in table and the finding of the overall (pooled) result was synthesized and summarized by using a forest plot. The data extracted in the Microsoft Excel sheet format was exported into the STATA/SE14 version statistical software for further analysis. Before estimating the pooled magnitude of disclosing HIV positive status, the heterogeneity among the selected study results was statistically estimated by using the I^2^ test. The pooled effect of the point estimate of disclosing HIV positive status in Ethiopia was calculated by DerSimonian & Liard’s method of random effect model at P-value less than 0.05 [40]. Statistical significance for heterogeneity with I^2^ tests greater than 75% was taken as high heterogeneity and it was subjected to sub-group and sensitivity analysis. Finally, publication bias was assessed by using Egger’s weighted regression test method and a p value less than 0.05 was considered as statistically significant publication bias [41].

## Results

### Study selection

**Fig 1:**
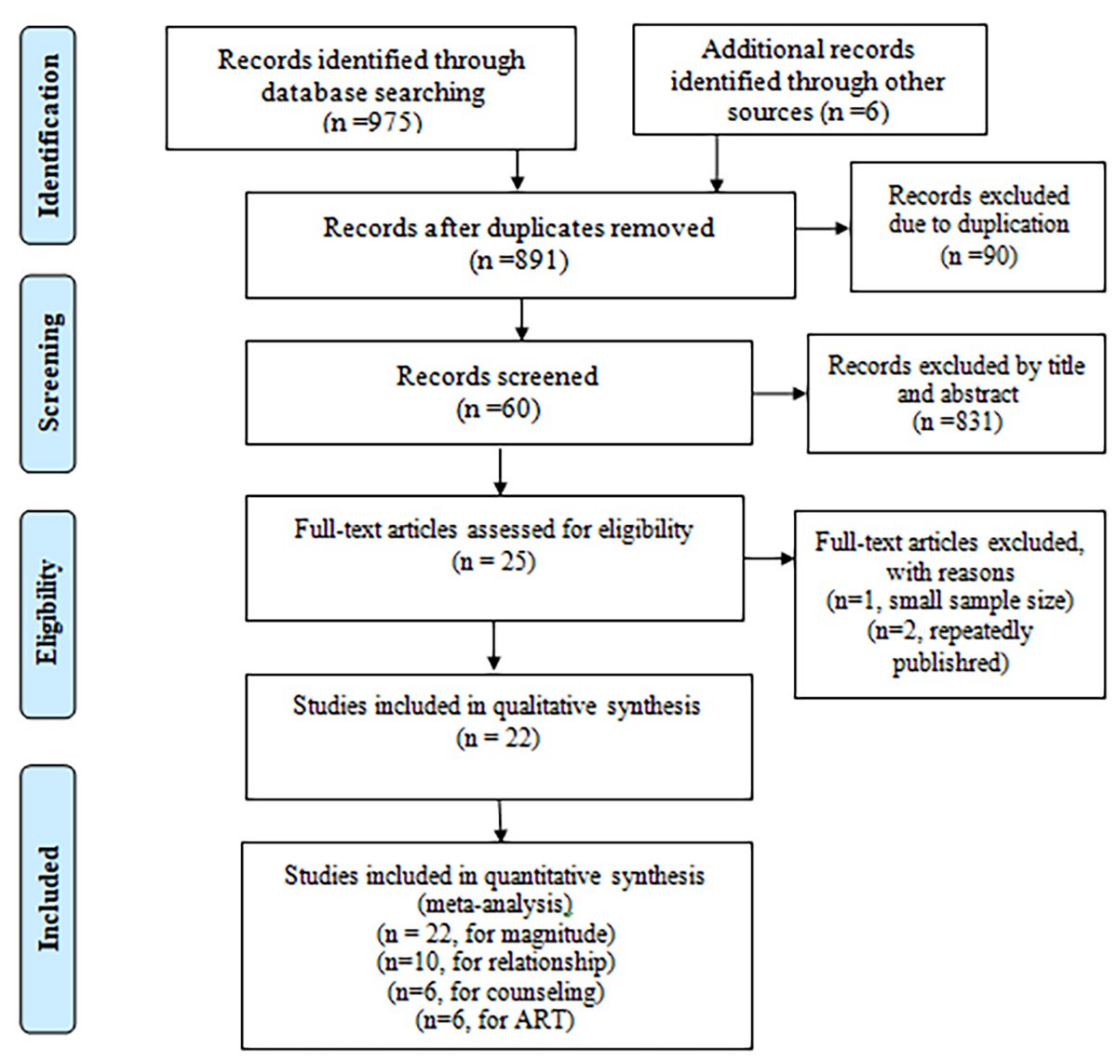
PRISMA flow diagram of determinants of disclosing HIV positive status to sexual partner among adult HIV patients in Ethiopia, 2020.

Several databases: PubMed, CINAHL, HINARI, Cochrane Library, Global Health and Google Scholar were used to search articles. The review found a total of 981 articles and nine hundred twenty-one of them were excluded (90 due to duplication and 831 records by title and abstract). Again 25 of them were critically appraised for eligibility based on the JBI checklist. Three of them were excluded due to reasons and a total of 22 full-text articles were included in systematic review and meta-analysis (Fig 1).

### Descriptive characteristics of included studies

All included articles were health facility-based cross-sectional studies conducted among adult HIV positive peoples in Ethiopia. The minimum and the maximum sample size were 107 and 705 participants in a study conducted in Addis Ababa and Jimma, Oromia respectively [23,42]. In this analysis, a total of 8, 873 adult HIV infected peoples were included. More than one-third (40.91%) of the studies were conducted in the Oromia region [23,26–30,43–45] and 54.55% of the studies were conducted only in the hospital [22–33]. Equal numbers of studies (four studies in each) were included in Addis Ababa [22,46–48] and Amhara region [33,49–51]. Whereas, only three articles conducted in Tigray region were included in the analysis [24,32,52] (Table 1).

**Table 1:** Characteristics of studies included in systematic review and meta-analysis.

Authors	Study year	Response Rate	Regions	Sex	Study Setting	Magnitude	Quality score
Geremew T et al	2016	100	Oromia	Both	Hospital	52.55	6
Erku T et al	2010	100	Amhara	Both	Hospital	76.65	7
Mussie A et a	2013	100	Tigray	Women	Hospital	63.81	8
Kassahun G et al	2017	99.7	Oromia	Women	Both	86.05	7
Dessalegn N et al	2015	100	Addis Ababa	Both	Hospital	82.54	8
Wasie B et al	2009	100	Amhara	Both	Health center	98.61	6
Deribe B et al	2017	100	SNNP	Women	Hospital	72.95	7
Koyira A	2009	94.7	Addis Ababa	Both	Both	88.27	6
Sendo E et al	2011	95.5	Addis Ababa	Women	Health center	72.90	7
Reda A et al	2010	-	Oromia	Both	Hospital	66.34	6
Gedisa T et al	2013	-	Oromia	Both	Hospital	91.11	7
Deribe K et al	2007	99.8	Oromia	Both	Hospital	90.78	7
Meseret Y et al	2016	99	Addis Ababa	Women	Health center	51.73	8
Alema H et al	2013	99.7	Tigray	Both	Both	41.83	8
Natae S et al	2013	99.5	Oromia	-	Hospital	84.92	6
Genet M T et al	2012	100	Tigray	Both	Hospital	57.41	7
Gari T et al	2008	100	SNNP	Women	Hospital	85.68	6
Shiferaw M et al	2019	100	Amhara	Both	Both	73.40	7
Fituma F et al	2012	99.5	Oromia	Both	Both	84.87	8
Tesfaye T et al	2014	98.1	Oromia	Both	Hospital	33.33	7
Fekadu H et al	2013	100	Oromia	Both	Both	94.14	6
Alemayehu D et al	2015	100	Amhara	Women	Both	89.73	8

SNNP-Southern Nation, Nationalities and Peoples.

### Magnitude of disclosing HIV positive status to sexual partner

**Fig 2:**
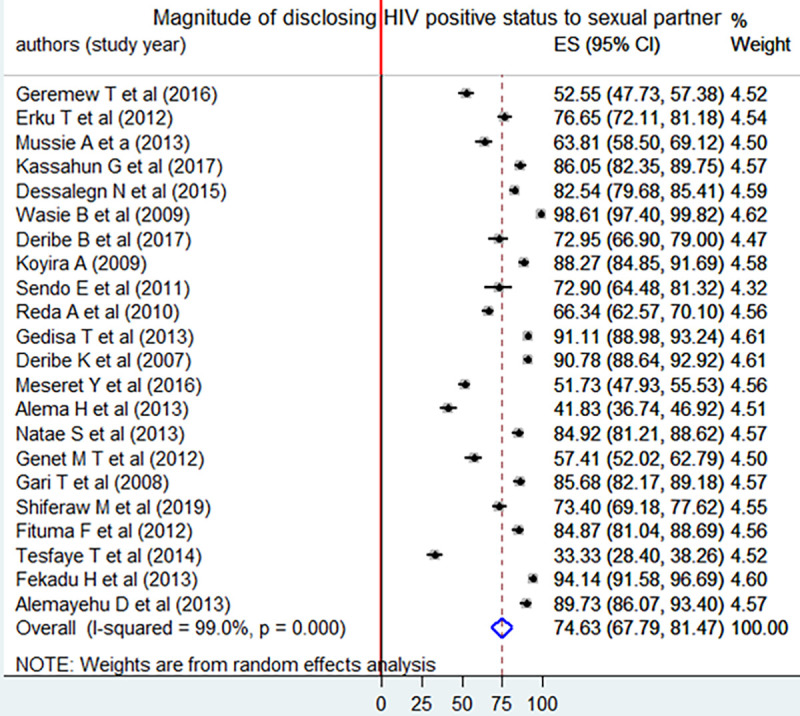
Forest plot of the pooled magnitude of disclosing HIV status to sexual partner using the random effect model, a systematic review and meta-analysis, Ethiopia, 2020.

The magnitude of disclosing HIV serostatus to sexual partner was a range between 33.33 to 98.6% in previous individual studies [30,49]. The I^2^ test result showed that there was high heterogeneity (I^2^ 99.0%, P < 0.001). Using the random effect analysis, the pooled magnitude of disclosing seropositive status to sexual partner among adult HIV peoples was 74.63% [95% CI: (67.79, 81.47)] (Fig 2).

**Fig 3:**
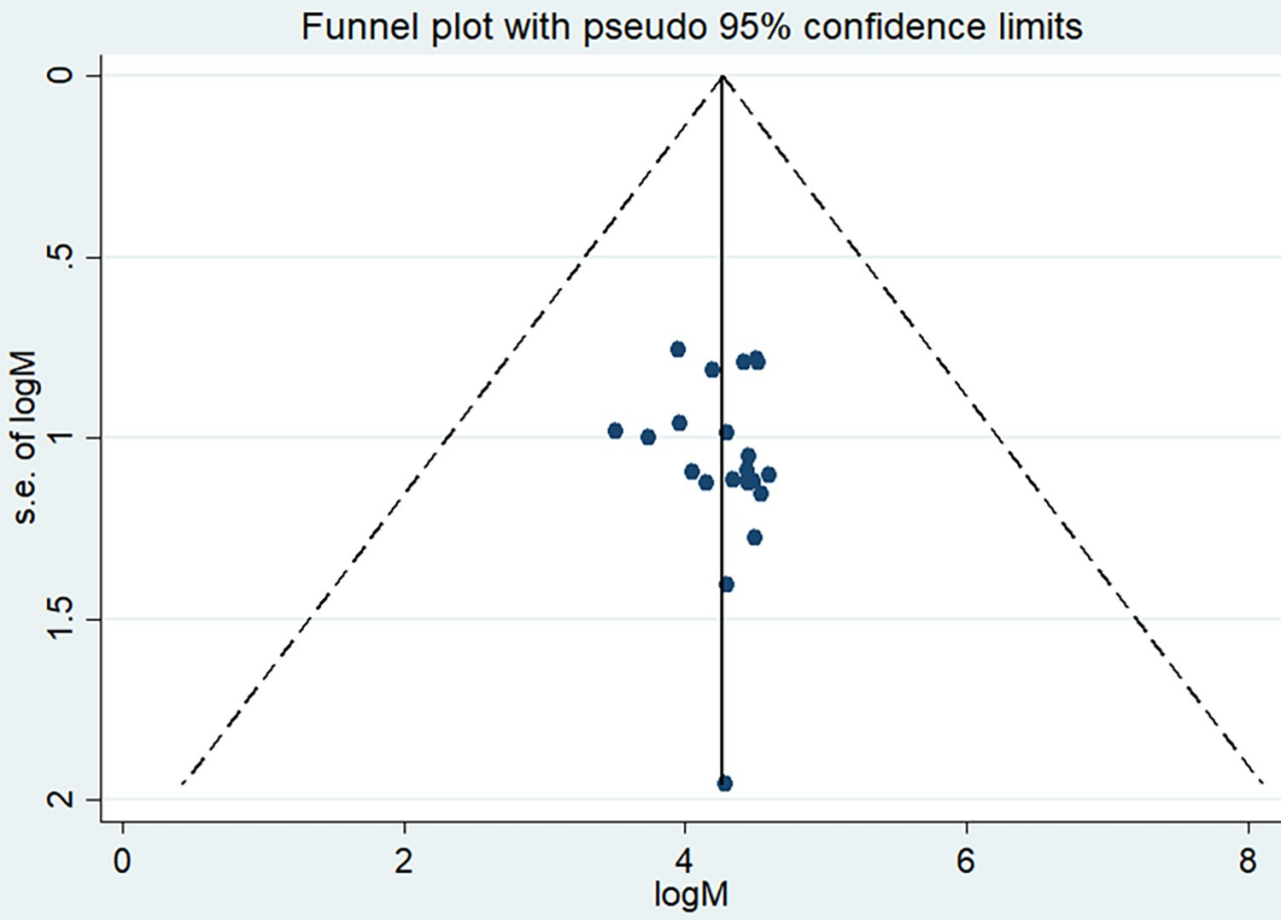
Funnel plot showing publication bias of magnitude of disclosing HIV status among adult HIV infected peoples, a systematic review and meta-analysis, Ethiopia, 2020.

Even though the funnel plot seems asymmetric (Fig 3), the Egger test revealed that there was no statistical evidence of publication bias (P = 0.629).

The sensitivity analysis also indicated that there was no single influential estimate that could be attributed to the source of heterogeneity.

### Subgroup analysis

**Fig 4:**
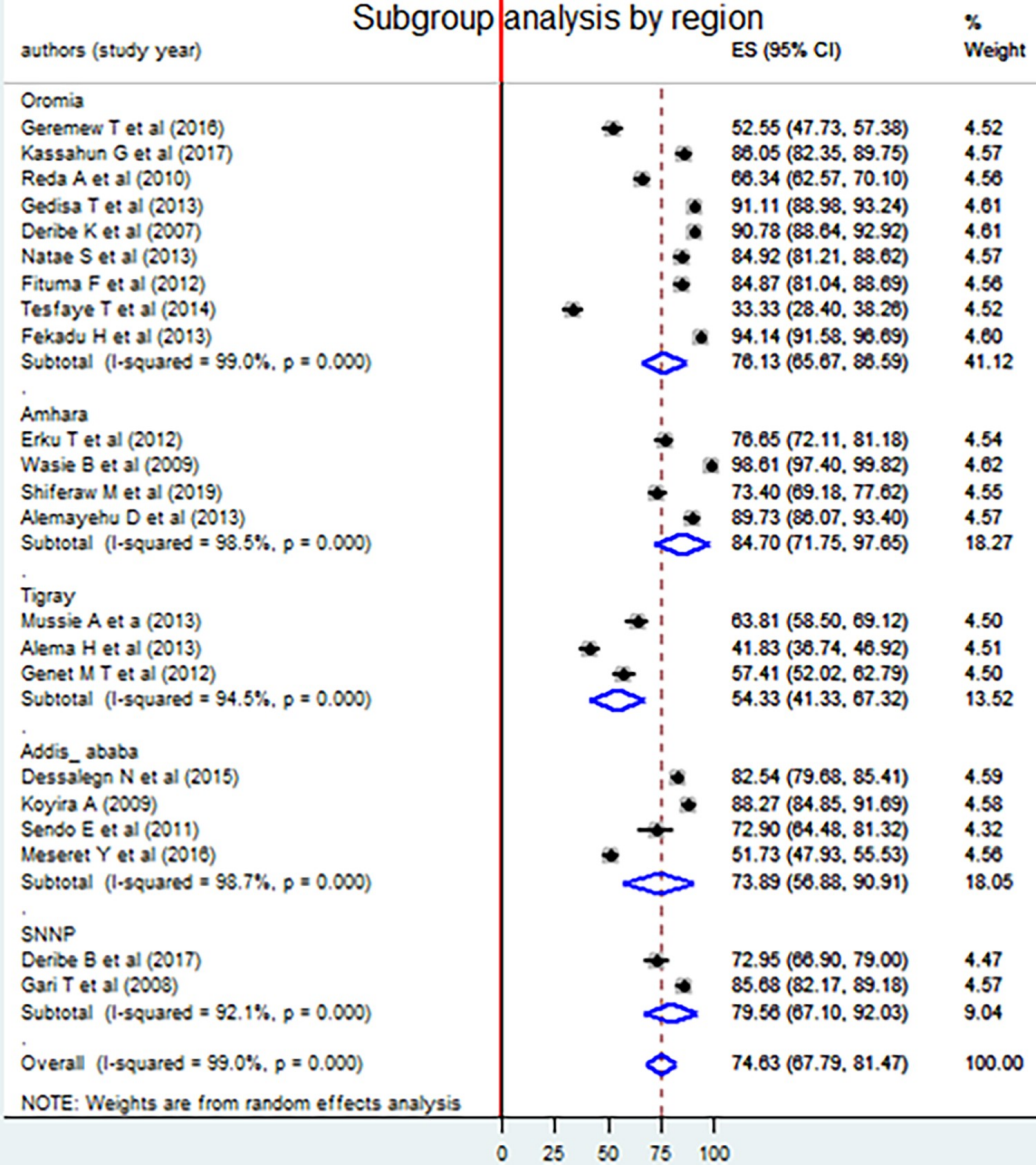
Forest plot of subgroup analysis by region among adult HIV infected peoples using the random effect model, a systematic review and meta-analysis, Ethiopia, 2020.

Subgroup analysis was done by region, sex of participants, study setting, sample size, year of study, quality score and response rate to deal the possible sources of heterogeneity. The analysis indicated that heterogeneity still exists in the subgroup analysis of all the parameters mentioned above. The highest and the lowest magnitude of disclosing HIV positive status to sexual partner was observed in Amhara [84.70%, 95% CI: (71.75, 97.65)] and Tigray [54.33%, 95% CI: (41.33, 67.32)] respectively (Fig 4).

### Factors associated with disclosing HIV status to sexual partner

**Fig 5:**
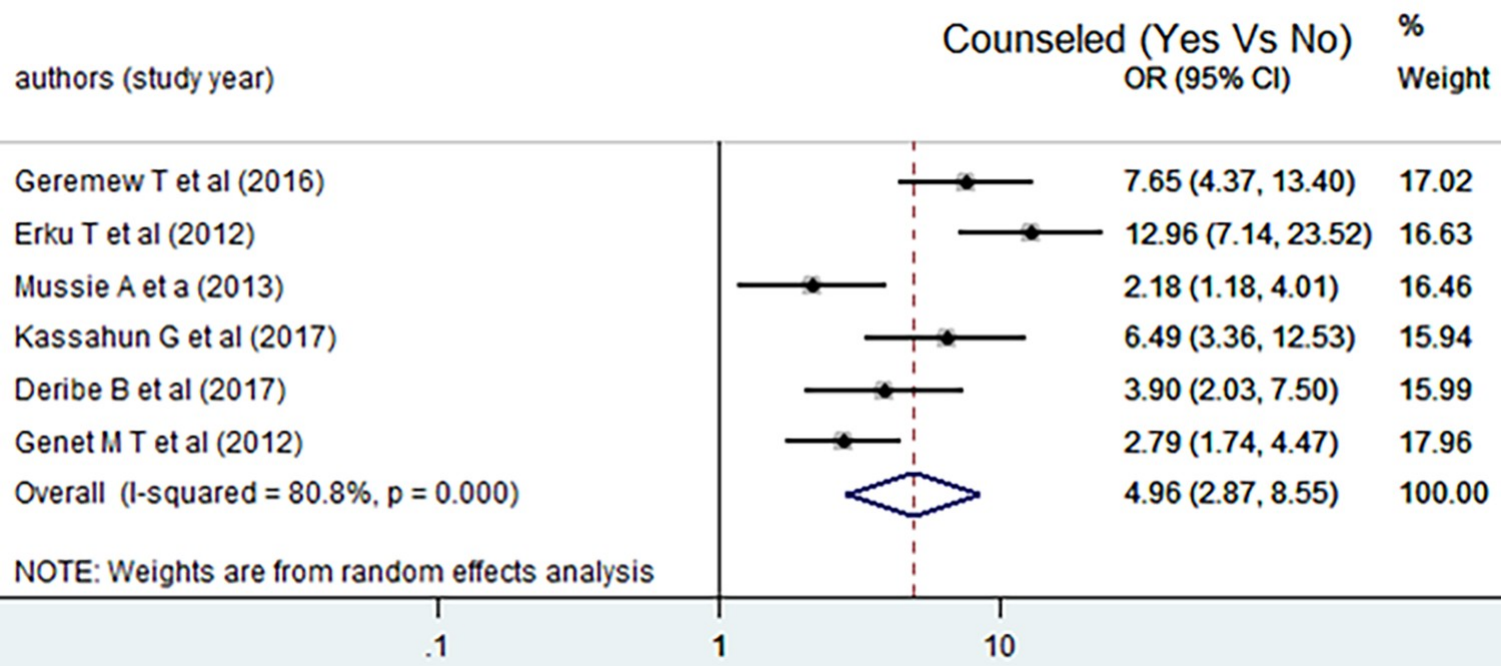
Forest plot of the pooled estimate of the effect of counseling on disclosing HIV positive status to sexual partner, a systematic review and meta-analysis, Ethiopia, 2020.

The analysis indicated that counseling, relationship before HIV testing and ART initiation was significantly associated with disclosing HIV to sexual partner. In the random effect model, the pooled odds of disclosing HIV positive status to sexual partner among counseled HIV infected adult individuals were increased by 5 as compared to counterparts [AOR = 4.96, 95% CI: (2.87, 8.55)]. As it is illustrated in the figure, all studies were contributed almost the same weight in effect size estimation. There was also considerable heterogeneity in those studies used to measure the pooled effect size (I^2^ = 80.8%, p < 0.001) (Fig 5).

**Fig 6:**
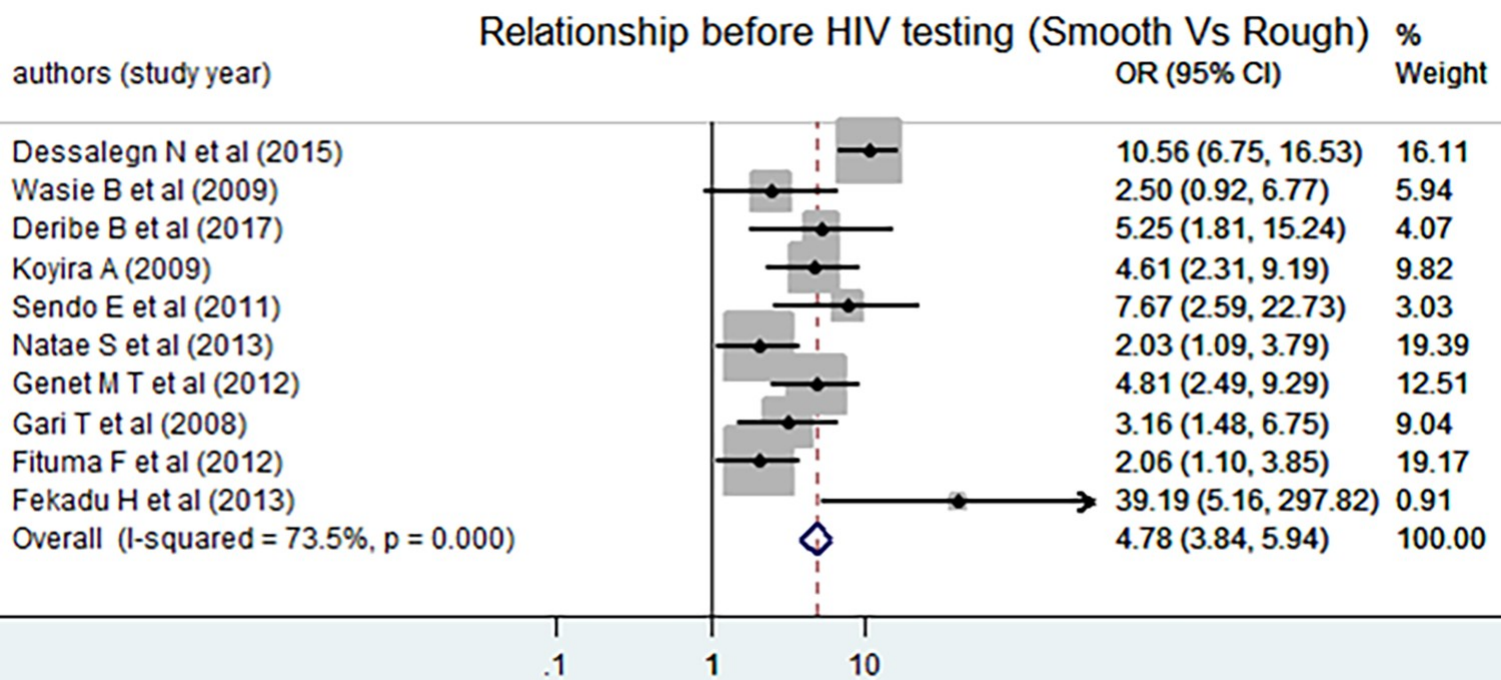
Forest plot of the pooled estimate of the effect of smooth relationship on disclosing HIV positive status to sexual partner, a systematic review and meta-analysis, Ethiopia, 2020.

The Egger test indicated that there was no evidence of publication bias (P = 0.550). As there was no evidence of heterogeneity, the fixed-effect model was used in determining the effect of relationship on disclosing HIV positive status to sexual partner. The pooled odds of disclosing HIV positive status to sexual partner among individuals who had a smooth relationship prior to HIV diagnosis were increased by 5 as compared counterparts [AOR = 4.78, 95% CI: (3.84, 5.94)]. There was a moderate level of heterogeneity (I^2^ = 73.5%) (Fig 6).

**Fig 7:**
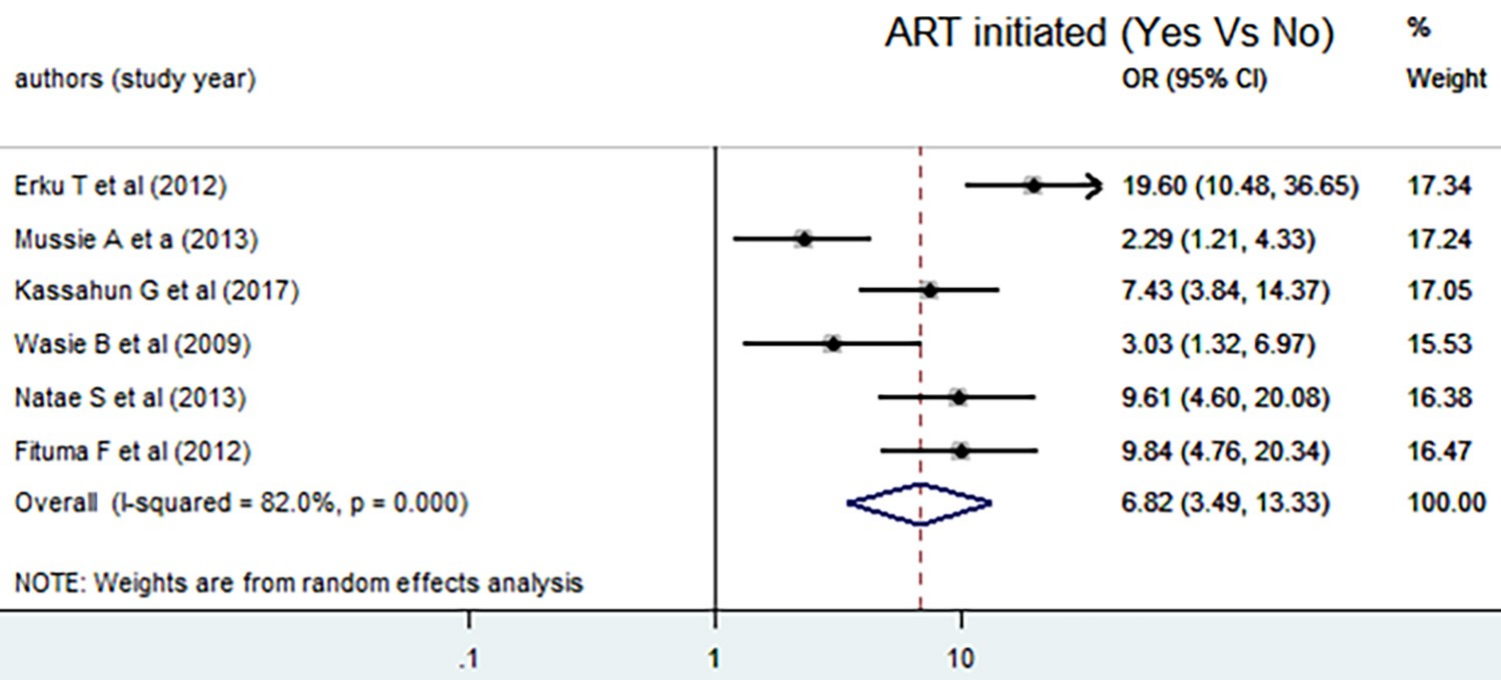
Forest plot of the pooled estimate of the effect of ART initiation on disclosing HIV positive status to sexual partner, a systematic review and meta-analysis, Ethiopia, 2020.

Similar to that of counseling, there was no evidence of publication bias (P = 0.963). In the random effect model, the pooled odds of disclosing HIV positive status to sexual partner among adult who had initiated ART were increased by 7 as compared to not initiated [AOR = 6.82, 95% CI: (3.49, 13.33)]. High heterogeneity was observed (I^2^ = 82.0%, P = 0.000) (Fig 7) and the Egger test statistics also witnessed that there was no evidence of publication bias (P = 0.649).

## Discussion

The result of meta-analysis showed that the pooled magnitude of disclosing HIV status to sexual partner was 74.63% [95% CI: (67.79, 81.47)]. ART initiation, counseling and type relationship before HIV diagnosis were significantly associated with disclosing HIV positive status to sexual partner. The pooled magnitude of disclosing HIV positive status to sexual partner was in line with a study conducted in Ethiopia (73%) [34]. It was also similar to studies conducted in Kenya (67.8%) and South Africa (74.4%) [8,53]. However, it was higher than studies conducted in Nigeria (50.9%), Uganda (57%) and South Africa (67%) [11,54,55]. Similarly, the finding was higher than a systematic review conducted in Sub-Saharan Africa (63.9%) [56]. The possible reason for this discrepancy may be due to time variation and the involvement of different governmental and non-governmental organizations in HIV prevention and control and thereby increase self-disclosure. Moreover, partner notification was one of the key emphasis for those organizations and still, it was under consideration for further HIV prevention [21]. Whereas, the finding was lower than another study conducted in Nigeria (87%) China (83.6%) and America (97%) [57–59]. The possible source of variation might be a difference in context and cultural practices as it was compared to studies conducted in developed countries.

The likelihood of disclosing HIV positive status to sexual partner was more among counseled adult HIV infected peoples than not. The report of a study conducted in Namibia showed that ever had HIV counseling positively associated with HIV disclosure [60]. It was also in agreement with a study conducted in Uganda [61]. The finding of systematic review in high and low-income countries also support this result which stated that those counseled were more likely to disclose their HIV status to sexual partner [62]. This association may be due to the fact that the counselor raises different issues and they may understand as there is a possibility of transmitting it to sexual partner even without intercourse.

The odds of disclosing HIV positive status to sexual partner among individuals who had smooth relationships prior to HIV diagnosis were increased as compared counterparts. A similar report was also documented in a study conducted in China [63]. The possible reason for this association might be due to lack of open discussion and honest and disguised practices seems enabled them to continue as sexual partner.

Those adult HIV infected peoples who had initiated ART were more likely to disclose HIV positive status to sexual partner as compared to pre-ART. It was in agreement with a study conducted in Uganda [64]. A study conducted in 6 countries of America witnessed that being in ART was positively associated with disclosing HIV positive status [65]. But, it was contrary to a study conducted in Namibia and Uganda [14,58]. The discrepancy may be due to the difference in socio-cultural practices and beliefs. The association could be due to observed improvement in their quality of life after treatment initiation and the health provider usually advised them to have one treatment supporter who would collect the drug in case of emergency and the clients may prefer their sexual partner as treatment collector instead of others.

As a limitation, the study was restricted to articles published in the English language and it may not be representative to articles published in a language other than English. The other demerit was as all the studies included in meta-analysis were cross-sectional; the pooled estimate may be influenced by study design. Lastly, even if subgroup analysis and sensitivity analysis was done to deal the source of heterogeneity, it was not resolved.

## Conclusions

Disclosing HIV positive status to sexual partner in Ethiopia was much low as effort were done on partner notification. Counseling, ART initiation and smooth relationship before HIV testing were significantly associated with disclosing HIV positive status to sexual partner. So, the government needs to strengthen pre and post HIV test counseling as well as continue it after treatment started. The universal test and treat strategy which was started recently should also another focus area of policymakers and planners to increase HIV disclosure. The last option falls on health care providers and HIV patients to advocate the role of trust and smooth relationships between partners through informed decision making.

## Supporting information

S1 File PRISMA checklist used in this systematic review and meta-analysis. (DOC)

S2 File The datasets used/analyzed in this systematic review and meta-analysis. (DTA)

## Abbreviations

AIDS: Acquired Immune Deficiency Syndrome
ART: Anti-retroviral Therapy
HIV: Human Immune Deficiency Virus
